# Studying the impact of young age on prognosis and treatment in laryngeal squamous cell carcinomas using the SEER database

**DOI:** 10.7717/peerj.7368

**Published:** 2019-07-25

**Authors:** Ruichen Li, Shitong Yu, Wenjia Zhu, Shengzi Wang, Li Yan

**Affiliations:** 1 Department of Radiation Oncology, Eye, Ear, Nose & Throat Hospital of Fudan University, Shanghai, China; 2 Department of General Surgery, Nanfang Hospital, Southern Medical University, Guangzhou, China; 3 Department of E.N.T, Eye, Ear, Nose & Throat Hospital of Fudan University, Shanghai, China

**Keywords:** SEER, Younger and older patients, Larynx preservation, Survival, Laryngeal cancer

## Abstract

**Background:**

Laryngeal squamous cell carcinoma (LSCC) in young patients was reported to be more aggressive and associated with poorer survival than in older patients. However, very few studies contained sufficient cases to permit meaningful statistical analysis. It is still unknown whether less aggressive method like radical radiotherapy (RT) is comparable to total laryngectomy (TL) in survival rate among young patients.

**Methods:**

This study extracted patient data from the surveillance, epidemiology, and end results database from 2004 to 2015. The findings for 304 patients (1.2%) below the age of 40 were compared with those for 24,827 patients (98.8%) aged 40 or older.

**Results:**

The younger cohorts contained a higher proportion of female patients (33.6% vs. 19.1%, respectively), had more cases of glottic involvement (64.5% vs. 53.1%), and were less likely to have distant metastasis (0.7% vs.3.2%). A total of 5-year overall survival and cancer-specific survival rates (CSS) in the younger patients were 86.3% and 88.8%, respectively, significantly better than for older patients (53.8% and 67.6%). Significant differences were still observed when stratified for tumor stage (stage I–IV). The negative independent prognostic factors in younger patients were advanced tumor stage, degree of nodal involvement, and status of distant metastasis. Treatment with surgery and/or RT all produced excellent outcomes in stage I–IV diseases, and radical RT resulted in survival rates equal to those for TL in locally advanced LSCC among young patients (5-year CSS: 90% vs. 91.5%, *p* = 0.99).

**Conclusion:**

LSCC is less aggressive and has significantly better survival in younger patients. For younger patients, advanced nodal involvement is the most important independent prognostic factor, and larynx preservation is comparable to TL in survival rate.

## Introduction

Laryngeal cancer is one of the most common tumors of the respiratory system. In 2019, the estimated incidence was 12,410 new cases, with approximately 3,760 patients dying from the disease (Siegel, Miller & Jemal, 2019). Laryngeal squamous cell carcinoma (LSCC) accounted for more than 95% of laryngeal cancers. It has been considered a cancer of older men with a history of tobacco use and alcohol ingestion (Muscat & Wynder, 1992; Steuer et al., 2017), with a peak age in the sixth decade (Misono et al., 2014). Of all patients with LSCC, less than 10% are younger than 40 (Misono et al., 2014; Nachalon et al., 2018; Singh et al., 2000). However, epidemiological data have shown that the incidence of head and neck squamous cell carcinoma (HNSCC) is rising in younger patients (Schantz & Yu, 2002; Schwartz, Hughes & Brigger, 2015). Since young people have a lower duration of exposure to the classic risk factors of tobacco and alcohol (Nachalon et al., 2018; Singh et al., 2000; Challapalli et al., 2018; Toporcov et al., 2015), LSCC in younger people may be due to other causes, for instance, the human papilloma virus, gene mutations, and family history of early-onset cancer (Toporcov et al., 2015; Ryu et al., 2019; Kreimer et al., 2005). As a consequence, a different disease etiology and behavior may predict different prognoses between the age cohorts.

Although age has a significant influence on the progression and development of cancer, its impact on survival outcomes remains controversial. Some studies have indicated that younger LSCC patients have poor survival rates, whereas other studies have suggested that age is not a prognostic factor (Nachalon et al., 2018; Singh et al., 2000; Reizenstein et al., 2010; Shvero et al., 1987). The inconclusive nature of these results may be attributed to limitations in sample size (Nachalon et al., 2018; Singh et al., 2000; Reizenstein et al., 2010; Shvero et al., 1987). Furthermore, many surgeons may be guided by their individual experience with older patients when making treatment decisions for younger patients, especially in the context of locally advanced cancer. A research focus on this special population is therefore crucial for determining the existence of survival differences under current treatment conditions because total laryngectomy (TL) deprives patients of the voice function and severely impacts their work and life.

Given the lack of consensus on whether risk factors, treatment methods, and prognosis for patients younger than 40 are the same as for older patients, we used the surveillance, epidemiology, and end results (SEER) tumor registry database to analyze how age influences treatment and survival outcomes. Our aim was to determine whether clinical characteristics differ between age groups and to provide a point of reference for treating younger patients, especially those with locally advanced cancer.

## Material and Methods

### Data collection

A retrospective study was conducted using the SEER database, which is publicly available. SEER data for 1973–2015 (year of diagnosis) were obtained via the SEER*Stat software (https://seer.cancer.gov). The selection process is provided in Fig. S1. All records were found at the following sites, using the site codes of the International Classification of Disease for Oncology, 3rd Edition (ICD-O-3): C32.0 (glottis), C32.1 (supraglottis), C32.2 (subglottis), C32.3 (laryngeal cartilage), C32.8 (overlapping lesion of larynx), and C32.9 (larynx, NOS). Exclusion criteria were tumor stage not specified, not SCC, without positive histology confirmation, not the first tumor, or survival information unknown. The tumor, node, and metastasis (TNM) stage was reclassified according to the eighth edition of the cancer staging system of the American Joint Committee on Cancer (AJCC). The study population who met the criteria was divided into younger and older groups (under 40, and 40 and over, respectively). The young patient (<40 years) was selected on the basis of our clinical observations, although its definition is consistent with previous reports in the literatures (Misono et al., 2014; Nachalon et al., 2018; Singh et al., 2000; Reizenstein et al., 2010; Shvero et al., 1987).

Clinical characteristics and treatment methods were included in the analysis, including age, gender, race, year of diagnosis, differentiated grade, primary site, stage of tumor, T status, N status, M status, surgery, radiotherapy (RT), chemotherapy, insurance, and marital status at the time of diagnosis. The LSCC cancer-specific survival (CSS) and non-CSS rates were extracted from the SEER variables of cause-specific death classification and other cause of death classification. The correlation between surgery and RT was extracted from the variables of radiation sequence with surgery, reason no cancer-directed surgery, and radiation recode. The type of surgery of the primary site was extracted from the variable of RX Summ-Surg Prim Site (1998+). According to SEER Program Coding and Staging Manual 2018, the code 40 (Total or radical laryngectomy, NOS), code 41 (TL ONLY), code 42 (Radical laryngectomy ONLY), and code 50 (Pharyngolaryngectomy) were defined as the coding for TL. Definitive radiation without surgery in locally advanced cancer was considered as larynx preservation (LP) in our study.

### Statistical analysis

Baseline clinical characteristics were compared using the Chi-squared test or Fisher’s exact test, as appropriate. The Kaplan–Meier method was used to estimate the probabilities of overall survival (OS) and CSS. The log-rank test and the Cox proportional hazards model were used for univariate and multivariate analysis, respectively. Statistical analyses were performed using SPSS, version 22 (IBM, Chicago, IL, USA). Variables with *p* < 0.05 in the univariate analyses were included in the multivariate analyses. For all analyses, *p*-values were two-sided, and *p* < 0.05 was considered statistically significant.

## Results

### Patient characteristics

**Figure 1:**
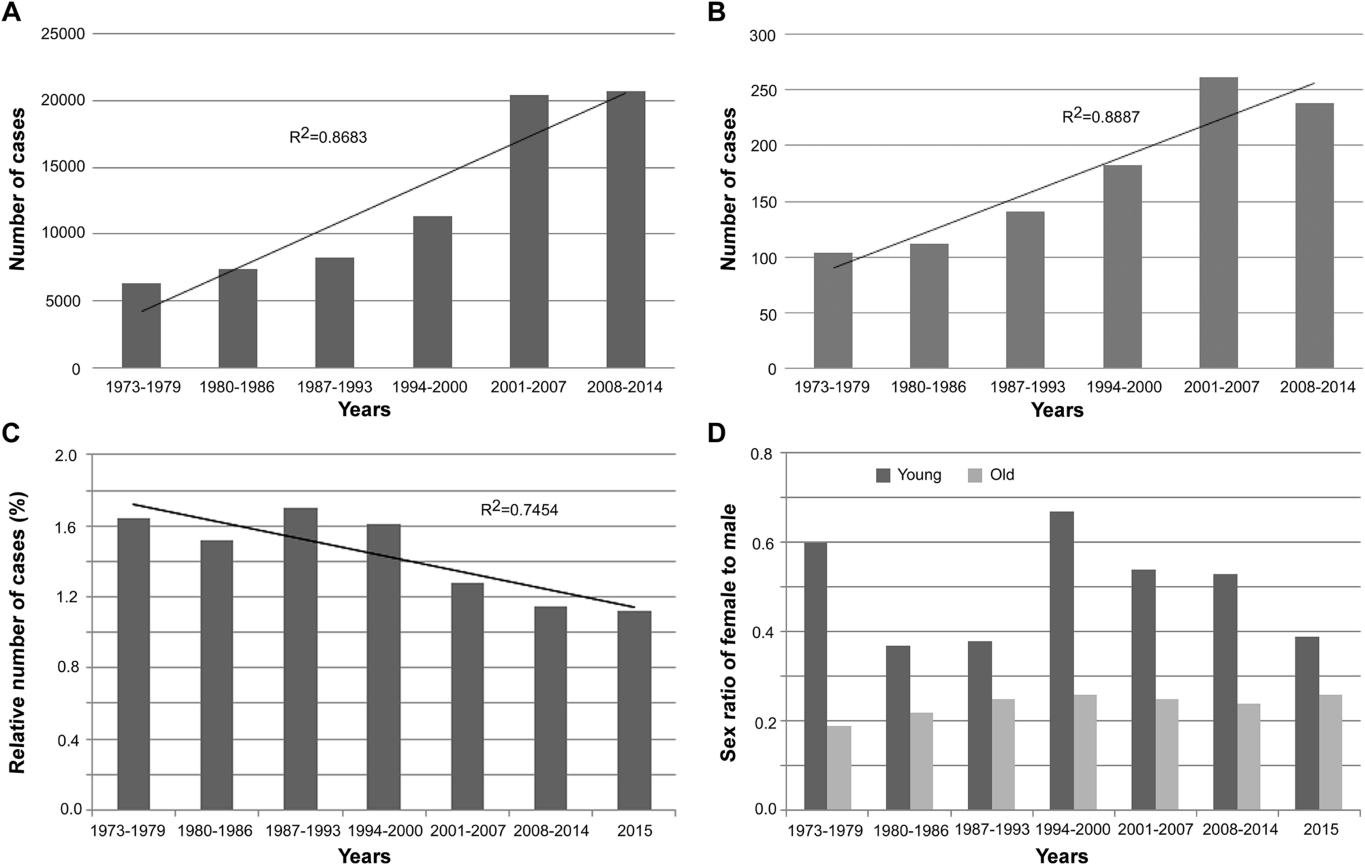
Laryngeal cancer statistics from 1973 to 2015 according to the SEER database. (A) Total number of new laryngeal cancer patients from 1973 to 2014. (B) Number of new laryngeal cancer patients aged <40 years from 1973 to 2014. (C) Number of new laryngeal cancer patients aged <40 years in proportion to the total number of laryngeal cancer cases from 1973 to 2015. (D) Sex ratio (female to male) of new laryngeal cancer patients stratified by age (40 years) from 1973 to 2015.

After a plateau during the period 1973–1993, there was a significant increase in the number of new cases of laryngeal cancer between 1994 and 2007. However, the growth rate had apparently declined since 2007 (Fig. 1A), and new cases of young patients were lower in 2008–2014 than in 2001–2007 (Fig. 1B). Moreover, the proportion of younger cases declined steadily between 1987 and 2015, reaching 1.12% in 2015 (Fig. 1C). The sex ratio (female to male) was higher in younger cohorts over the entire period from 1973 to 2015 (Fig. 1D).

As shown in Fig. S1, patients who were diagnosed in the period from 1973 to 2003 were excluded from the selection process, as their AJCC TNM stage status was unavailable. LSCC accounted for 97.1% (31,247 out of 32,169) of laryngeal cancer cases in the SEER database from 2004 to 2015. As a consequence, 25,131 patients were included in our study (304 aged <40 and 24,827 aged ≥40). The basic clinicopathological information is summarized in Table 1. The mean age of the 304 (1.2%) patients in the younger cohorts was 33.9 ± 5.938. The younger cohorts had higher proportions of female patients (33.6% vs. 19.1% for the older cohorts, *p* < 0.0001), glottic involvement (64.5% vs. 53.1%, *p* < 0.0001), treatment with surgery (54.3% vs. 40.2%, *p* < 0.0001) and treatment without RT (22.7% vs. 13.5%, *p* < 0.0001). The younger cohorts were less likely to have distant metastasis (0.7% vs. 3.2%, *p* = 0.005), were less likely to have insurance (62.5% vs. 70.8%, *p* = 0.002), and were less likely to be married (45.1% vs. 51.1%, *p* = 0.036). No significant differences were found between the groups in terms of race, differentiated grade, stage of disease, T status, N status, or treatment with or without chemotherapy.

**Table 1:** Clinical characteristics and treatment methods of evaluable patients stratified by age.

Variable	Age < 40 years No. (%)	Age ≥ 40 years No. (%)	*p*
Mean age ± SD	33.9 ± 5.938	64.12 ± 10.764	
Sex			<0.0001
Male	202 (66.4)	20,081 (80.9)	
Female	102 (33.6)	4,746 (19.1)	
Race			0.376
White	251 (82.6)	19,947 (80.3)	
Black	39 (12.8)	3,882 (15.6)	
Others	14 (4.6)	998 (4)	
Grade			0.231
Well differentiated	44 (14.5)	3,366 (13.6)	
Moderately differentiated	141 (46.4)	11,976 (48.2)	
Poorly or undifferentiated	47 (15.5)	4,591 (18.5)	
Unknown	72 (23.7)	4,894 (19.7)	
Site			<0.0001
Supraglottis	74 (24.3)	8,900 (35.8)	
Glottis	196 (64.5)	13,184 (53.1)	
Sublarynx	5 (1.6)	407 (1.6)	
Others	29 (9.5)	2,336 (9.4)	
Stage			0.658
I	110 (36.2)	9,011 (36.3)	
II	51 (16.8)	4,230 (17)	
III	61 (20.1)	4,350 (17.5)	
IV	82 (27)	7,236 (29.1)	
T status			0.372
T1	115 (37.8)	9,716 (39.1)	
T2	66 (21.7)	6,080 (24.5)	
T3	74 (24.3)	4,983 (20.1)	
T4	49 (16.1)	3,958 (15.9)	
Unknown	0 (0)	90 (0.4)	
N status			0.396
N0	225 (74)	17,761 (71.5)	
N1	24 (7.9)	2,403 (9.7)	
N2	49 (16.1)	4,187 (16.9)	
N3	3 (1)	368 (1.5)	
Unknown	3 (1)	108 (0.4)	
M status			0.005
M0	298 (98)	23,895 (96.2)	
M1	2 (0.7)	783 (3.2)	
Unknown	4 (1.3)	149 (0.6)	
Surgery			<0.0001
Yes	165 (54.3)	9,974 (40.2)	
No	137 (45.1)	14,754 (59.4)	
Unknown	2 (0.7)	99 (0.4)	
Radiotherapy			<0.0001
Yes	219 (72)	19,278 (77.6)	
No	69 (22.7)	3,364 (13.5)	
Unknown	16 (5.3)	2,185 (8.8)	
Chemotherapy			0.142
Yes	116 (38.2)	8,476 (34.1)	
No/unknown	188 (61.8)	16,351 (65.9)	
Insurance status at diagnosis			0.002
Any	190 (62.5)	17,575 (70.8)	
None or unknown	114 (37.5)	7,252 (29.2)	
Marital status at diagnosis			0.036
Any	137 (45.1)	12,692 (51.1)	
None or unknown	167 (54.9)	12,135 (48.9)	

### Survival analysis

**Figure 2:**
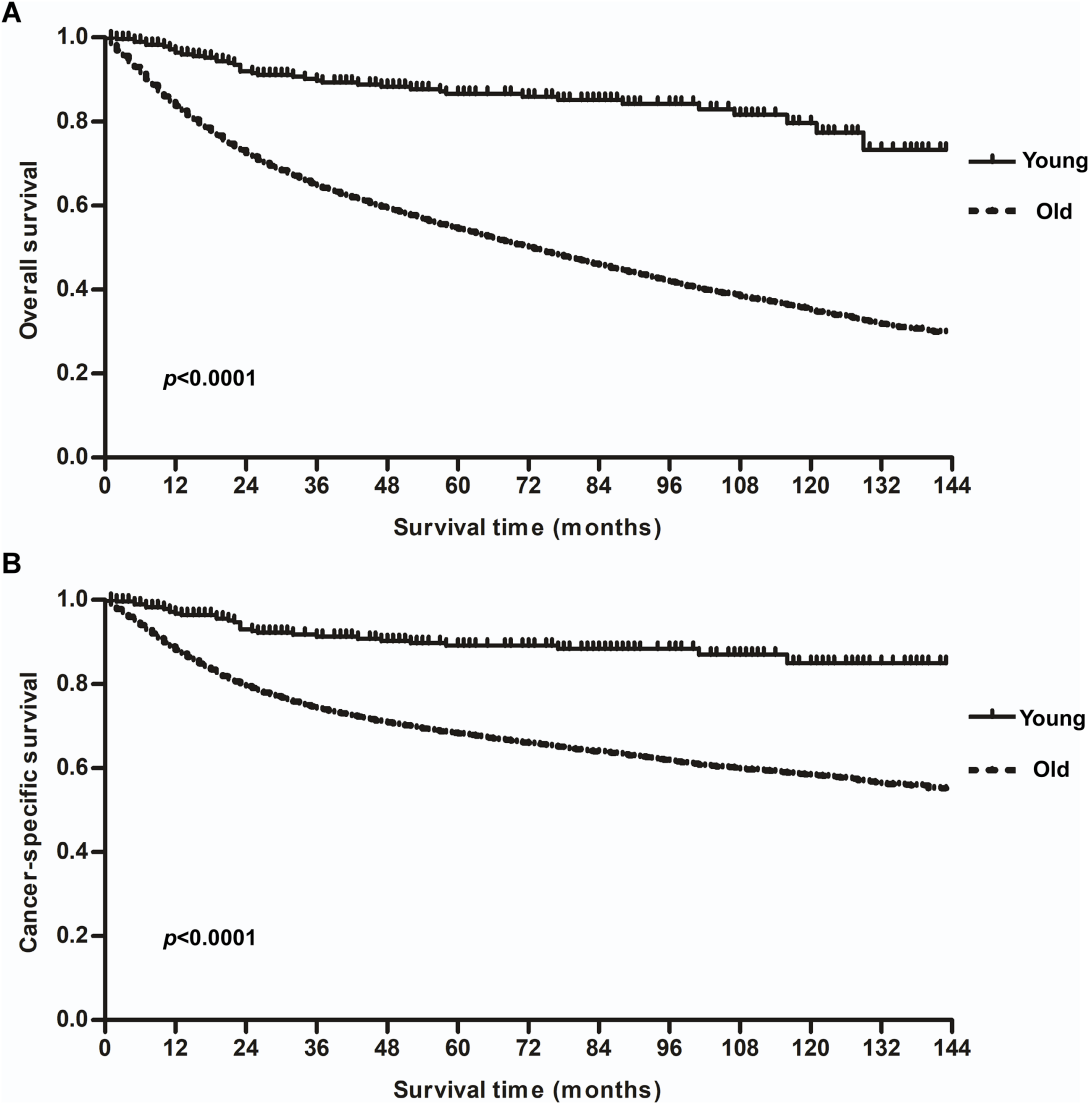
Kaplan–Meier overall survival (OS) and cancer-specific survival (CSS) curves for patients with laryngeal squamous cell carcinoma stratified by age (40 years). (A) OS of younger vs. older patients. (B) CSS of younger vs. older patients.

**Figure 3:**
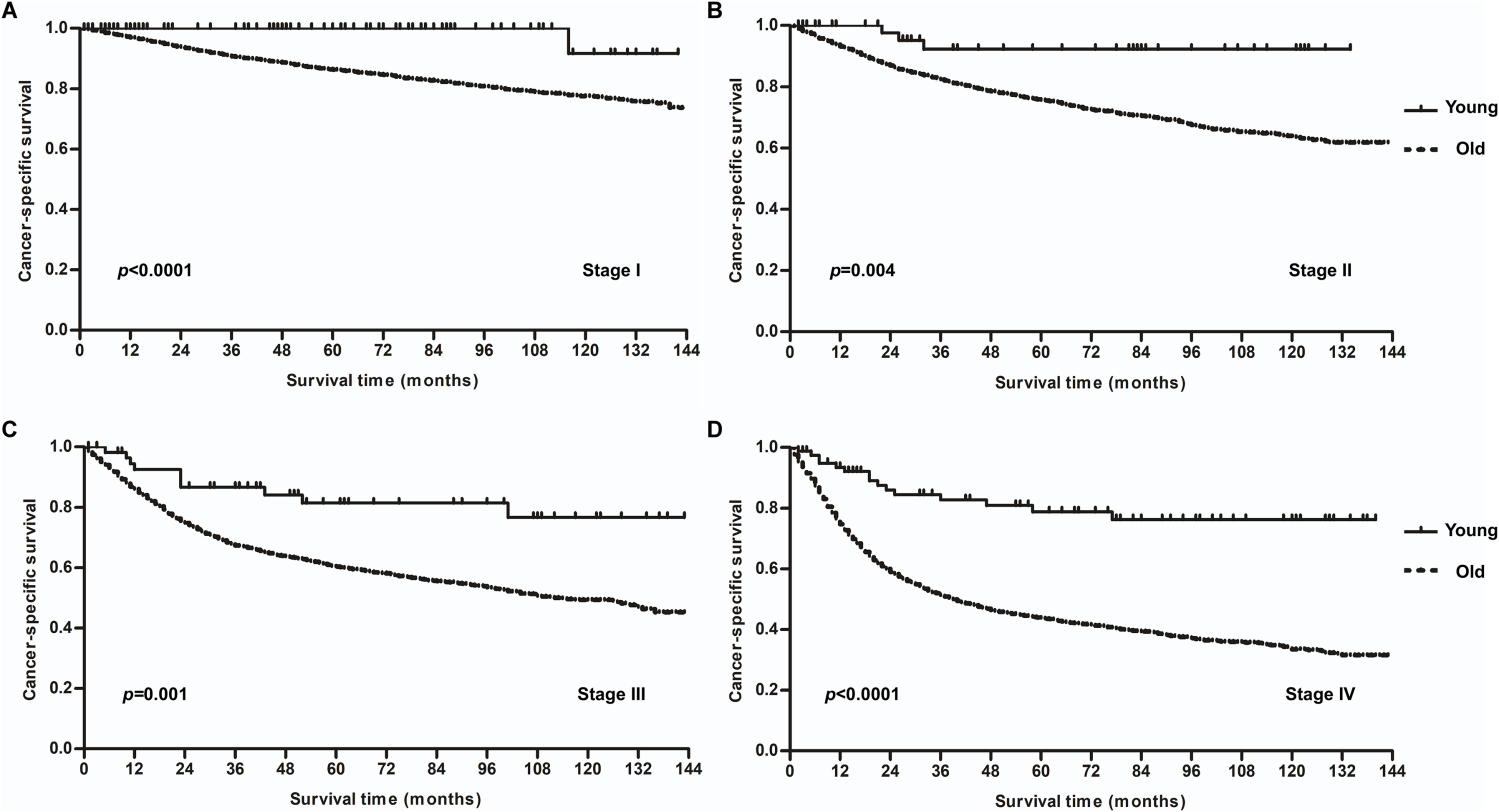
Cancer-specific survival (CSS) curves for patients with laryngeal squamous cell carcinoma stratified by age (40 years) at different tumor stages. (A) CSS of younger vs. older patients at stage I. (B) CSS of younger vs. older patients at stage II. (C) CSS of younger vs. older patients at stage III. (D) CSS of younger vs. older patients at stage IV.

The survival outcomes for the two cohorts are shown in Fig. 2. Patients in the younger group had a distinctly better survival rate than those in the older group. The 5-year OS and CSS in the younger group were 86.3% and 88.8%, respectively, compared to 53.8% and 67.6% in the older group (*p* < 0.0001). When matched for tumor stage, significant differences in the survival of patients with stage I–IV diseases were observed between the groups (Fig. 3). The younger patients showed better outcomes than the older patients at every stage (*p* < 0.05). The 5-year CSS rate for the young patients at stage I, II, III, IV was 100%, 92.2%, 81.4%, and 77.9%, respectively. Interesting, there were no significant difference between I and II (*p* = 0.069), II and III (*p* = 0.102) or III and IV (*p* = 0.628).

**Figure 4:**
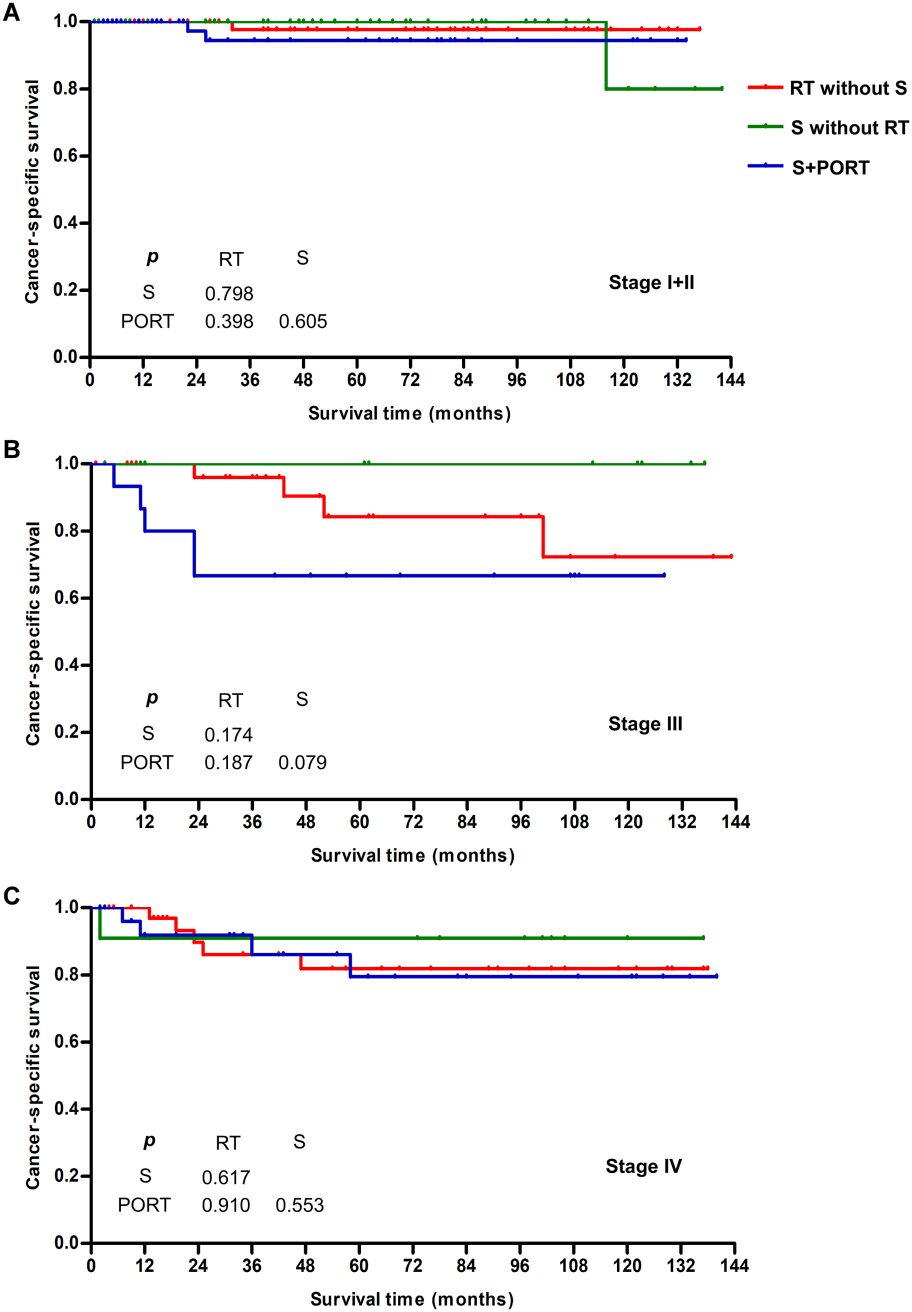
Survival curves for various treatment modalities at different tumor stages for patients <40 years old. (A) Stages I and II. (B) Stage III. (C) Stage IV. Abbreviations: RT, radiotherapy; S, surgery; PORT, postoperative radiotherapy.

**Figure 5:**
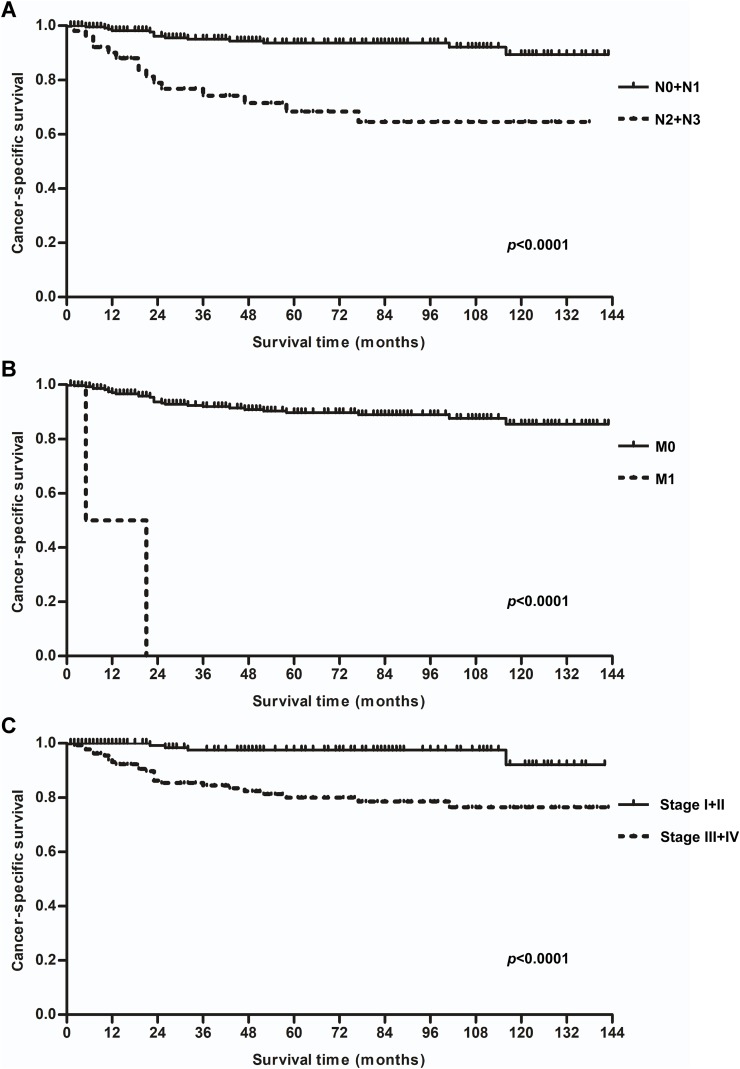
Cancer-specific survival for larynx preservation radiotherapy and total laryngectomy in locally advanced cancer without distant metastasis (T4N0–3M0). (A) Patients younger than 40. (B) Patients aged 40 or older. Abbreviations: RT, radiotherapy; S, surgery; TL, total laryngectomy.

The multivariate analysis for the whole cohort had been described in Table S1. Overall, patients aged <40 had higher cancer-specific mortality risk than aged ≥40 (hazard ratio [HR] = 3.579, *p* < 0.0001). We went on to examine the correlation between survival and other parameters stratified by age. In the younger group, univariate analysis revealed that tumor site (*p* < 0.0001), TNM stage (*p* < 0.0001), T status (*p* = 0.002), N status (*p* < 0.0001), M status (*p* < 0.0001), and use of chemotherapy (*p* < 0.0001) significantly affected the prognosis, as shown in Table 2. Surprisingly, no significant difference was found for differentiated grade (*p* = 0.271), treatment with surgery (*p* = 0.638), or treatment with RT (*p* = 0.15). To characterize better the impact of treatment methods on survival of younger patients, we divided the correlation between surgery and RT according to four levels of treatment: RT without surgery (*n* = 122), surgery without RT (*n* = 68), surgery combined with postoperative radiation (*n* = 91), and other of unknown sequences (*n* = 23). After matching for tumor stage (at which point 23 cases of other or unknown sequences were excluded), no significant differences in the survival of patients with stage I–IV diseases were observed between the three levels (Fig. 4). Treatment with surgery and/or RT produced excellent survival outcomes. In a comparison of survival outcomes between TL and radical RT without surgery (RT) in locally advanced cancer (T4N0–3M0), a total of 14 patients received RT, and 24 patients received TL. The 5-year CSS was 90% for RT and 91.5% for TL, with no significant difference (*p* = 0.99), as shown in Fig. 5A.

**Table 2:** Univariate analysis predicting cancer-specific survival (CSS) stratified by age.

Variable	Age < 40 years		Age ≥ 40 years	
	5-year CSS (%)	*p*	5-year CSS (%)	*p*
Sex		0.336		0.008
Male	87.4		67.9	
Female	91.6		66.3	
Race		0.454		<0.0001
White	88.4		68.8	
Black	93.9		60.3	
Others	81.5		71.7	
Grade		0.271		<0.0001
Well differentiated	94.3		79.6	
Moderately differentiated	86.9		67.9	
Poorly or undifferentiated	85.6		53	
Unknown	91		72.2	
Site		<0.0001		<0.0001
Supraglottis	77.9		55.4	
Glottis	93.8		79.2	
Others	87.3		49.2	
Stage		<0.0001		<0.0001
Early (I+II)	97.5		82.8	
Late (III+IV)	79.5		49.4	
T status		0.002		<0.0001
Early (T1+T2)	94.2		77.6	
Late (T3+T4)	81		49.6	
N status		<0.0001		<0.0001
Early (N0+N1)	93.7		73.8	
Late (N2+N3)	68.4		40	
M status		<0.0001		<0.0001
M0	89.7		69.6	
M1	0		15.7	
Surgery		0.638		<0.0001
Yes	90.2		71.7	
No	87.1		64.9	
Radiotherapy		0.15		<0.0001
Yes	87.8		69	
No	96.9		75.3	
Chemotherapy		<0.0001		<0.0001
No or unknown	95.9		74.3	
Yes	78.1		54.8	
Insurance status at diagnosis		0.471		0.087
None or unknown	90.2		67	
Any	87.7		67.9	
Marital status at diagnosis		0.239		<0.0001
None or unknown	85.9		60.7	
Any	92.1		73.8	

**Figure 6:**
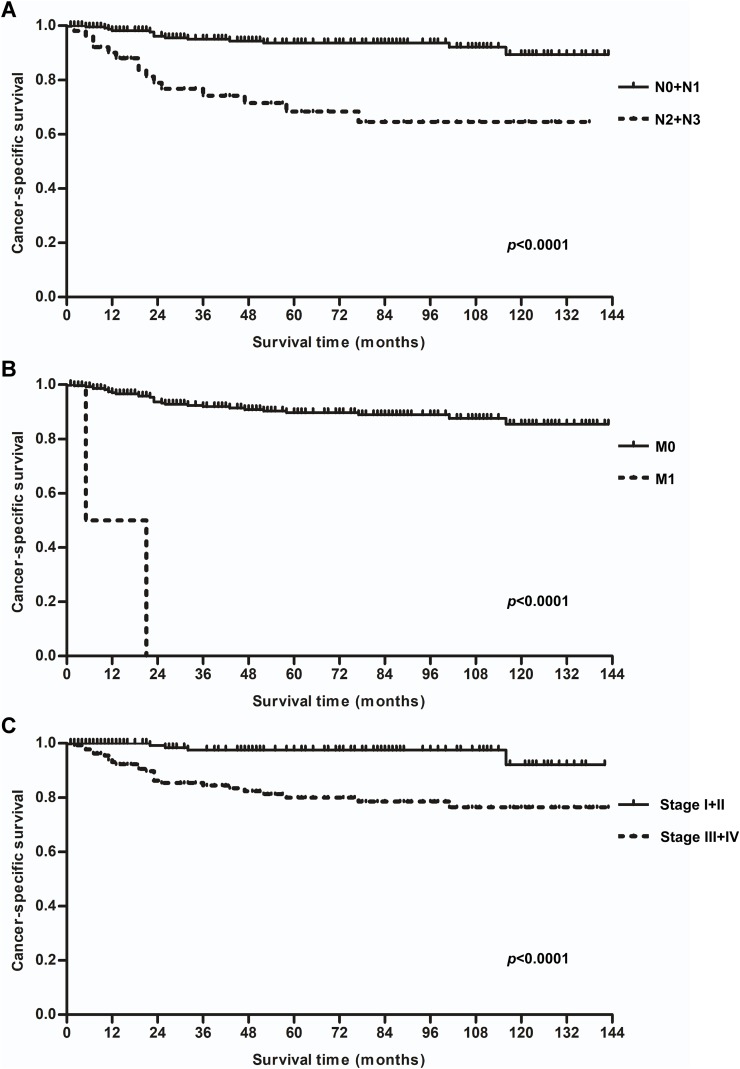
Kaplan–Meier cancer-specific survival rates for laryngeal squamous cell carcinoma patients aged younger than 40. (A) Nodal involvement. (B) Status of distant metastasis. (C) Tumor stage.

In the multivariate analyses (Table 3), stage III + stage IV (HR = 4.505, *p* = 0.011), N2 + N3 (HR = 2.525, *p* = 0.028), and M1 (HR = 12.704, *p* = 0.001) were significantly poor independent prognostic factors for CSS. As Fig. 6 shows, patients with early N stage (N0 + N1) had a 5-year CSS of 93.7%, which fell to 68.4% with advanced N stage (N2 + N3). Similarly, there were significant differences in the survival curves of patients with or without distant metastasis (*p* < 0.0001) and advanced or early tumor stage (*p* < 0.0001). Given that only two patients had distant metastasis (Table 1), degree of lymph node involvement seems to be the most important independent prognostic factor among the younger cohorts. As shown in Table 4, patients with advanced N stage (N2 + N3) had higher proportions of poorly differentiated or undifferentiated tumors (28.8% vs. 12.4% for the advanced and early stages, respectively, *p* = 0.007) and supraglottic involvement (65.4% vs. 16.1%, *p* < 0.0001). They were also more likely to be treated with RT (88.5% vs. 69.1%, *p* = 0.002) and chemotherapy (84.6% vs. 28.5%, *p* < 0.0001), and they were less likely to be treated with surgery (36.5% vs. 57.8%, *p* = 0.012).

**Table 3:** Multivariate Cox regression analysis predicting cancer-specific survival (CSS) stratified by age.

Variable	Age < 40 years		Age ≥ 40 years	
	HR (95% CI)	*p*	HR (95% CI)	*p*
Sex	Not included			<0.0001
Female/Male			0.844 [0.79–0.902]	
Race	Not included			
Black/White				0.59
Others/Black				0.301
Grade	Not included			
Moderately/Well			1.265 [1.151–1.391]	<0.0001
Poorly or undifferentiated/Well			1.526 [1.377–1.692]	<0.0001
Unknown/Well			1.174 [1.054–1.307]	0.003
Site				
Glottis/Supraglottis		0.436	0.702 [0.656–0.751]	<0.0001
Others/Supraglottis		0.843	1.177 [1.088–1.273]	<0.0001
Stage				
Late (III+IV)/Early (I+II)	4.505 [1.412–14.379]	0.011	1.94 [1.753–2.147]	<0.0001
T status				
Late (T3+T4)/Early (T1+T2)		0.635	1.247 [1.153–1.349]	<0.0001
N status				
Late (N2+N3)/Early (N0+N1)	2.525 [1.106–5.762]	0.028	1.552 [1.451–1.659]	<0.0001
M status				
M1/M0	12.704 [2.668–60.485]	0.001	2.649 [2.361–2.972]	<0.0001
Surgery	Not included			
No/Yes			1.29 [1.215–1.37]	<0.0001
Radiotherapy	Not included			
No/Yes			1.124 [1.027–1.23]	0.011
Chemotherapy				
Yes/No or unknown		0.139	0.883 [0.825–0.946]	<0.0001
Insurance status at diagnosis	Not included		Not included	
Any/None or unknown				
Marital status at diagnosis	Not included			
Any/None or unknown			0.746 [0.708–0.786]	<0.0001

**Note:**

HR, hazard ratio; CI, confidence interval.

**Table 4:** Characteristics of patients younger than 40 years stratified by lymph node status (*N*).

Variable	N0+N1 No. (%)	N2+N3 No. (%)	*p*
Grade			0.007
Well differentiated	41 (16.5)	3 (5.8)	
Moderately differentiated	115 (46.2)	25 (48.1)	
Poorly or undifferentiated	31 (12.4)	15 (28.8)	
Unknown	62 (24.9)	9 (17.3)	
Site			<0.0001
Supraglottis	40 (16.1)	34 (65.4)	
Glottis	185 (74.3)	11 (21.2)	
Others	24 (9.6)	7 (13.5)	
Surgery			0.012
Yes	144 (57.8)	19 (36.5)	
No	103 (41.4)	33 (63.5)	
Unknown	2 (0.8)	0 (0)	
Radiotherapy			0.002
Yes	172 (69.1)	46 (88.5)	
No	65 (26.1)	3 (5.8)	
Unknown	12 (4.8)	3 (5.8)	
Chemotherapy			<0.0001
Yes	71 (28.5)	44 (84.6)	
No/unknown	178 (71.5)	8 (15.4)	

In the older group, the univariate and multivariate analyses of factors predictive of CSS showed that the factors significantly correlated with survival outcomes were gender (*p* < 0.0001), differentiated grade (*p* < 0.0001), site (*p* < 0.0001), tumor stage (*p* < 0.0001), T status (*p* < 0.0001), N status (*p* < 0.0001), M status (*p* < 0.0001), surgery (*p* < 0.0001), RT (*p* = 0.011), chemotherapy (*p* < 0.0001), and marital status (*p* < 0.0001), as shown in Tables 2 and 3. Interestingly, certain factors that were not independent prognostic factors for the younger patients resulted in significantly better survival rates in the older patients: female sex, well-differentiated tumor, involvement of glottis, early T status, treatment with surgery, treatment with RT, use of chemotherapy, and being married. In locally advanced cancers (T4N0–3M0), a total of 1,086 patients received RT, and 1,679 patients received TL. Among the older patients, the analysis revealed significantly worse 5-year CSS after RT (42.3%) compared with TL (56%), as shown in Fig. 5B.

## Discussion

This study includes one of the largest cohorts of patients who were below the age of 40 when they were diagnosed with LSCC. We found significant differences in OS and CSS between younger and older patients. In addition, the younger cohort included a significantly higher proportion of women and tended to have less aggressive disease (in terms of M status) than the older group. Advanced nodal involvement (N2 + N3) seems to be the most important independent prognostic factor for younger patients. Furthermore, among young patients with locally advanced larynx cancer, the RT method resulted in survival rates equal to those for TL.

As reported in *CA: A Cancer Journal for Clinicians* (Steuer et al., 2017), laryngeal cancer is one of the few cancers in which 5-year OS has declined in recent decades, from 66% to 63%, although its incidence is declining, too. Indeed, in the 5 years from 2015 to 2019, the estimated numbers of new cases were 13,560, 13,430, 13,360, 13,150, and 12,410, respectively, (Siegel, Miller & Jemal, 2015, 2016, 2017, 2018, 2019). In our study based on the SEER database, we found that the growth rate had apparently declined since 2007. The decline in incidence may be attributed to broader education about the dangers of alcohol and tobacco. Many studies have proved that tobacco use and alcohol ingestion have a linear relationship with the development of laryngeal cancer (Muscat & Wynder, 1992; Steuer et al., 2017; Boffetta & Hashibe, 2006), and the heaviest smokers have a risk up to 30 times greater (Rothman et al., 1980; Kuper, Boffetta & Adami, 2002). However, Llewellyn et al. (2004) found that smoking for 21 years or more was necessary to increase the odds of acquiring a head and neck malignancy, with lower odds in those aged 45 or younger. This suggests that LSCC in the younger population may be due to causes other than tobacco and alcohol. Surprisingly, according to the SEER database in our study, not only did the proportion of younger cases steadily decline from 1987 to 2015, but also the number of new cases in young patients decreased between 2008 and 2014 when compared to 2001 and 2007 (Fig. 1). A rise in the number of older patients cause by the rapid increase in the elderly population may explain the decline in the proportion of younger cases. Nonetheless, there are limited data as to why the number of new cases in younger patients decreased at the same time, since tobacco- and alcohol-related risk factors may not be applicable to younger patients (Nachalon et al., 2018; Singh et al., 2000; Challapalli et al., 2018; Toporcov et al., 2015). The epidemiological data and previous studies all indicate that LSCC among younger patients may be a distinct disease (Challapalli et al., 2018).

A significant difference in the younger group was the relatively high proportion of female patients, whereas male patients were much more common among the older cohort. Lund & Howard (1990) have indicated an equalization of the male to female ratio in the younger HNSCC cohort, ranging from 1 to 2:1, which is consistent with our findings. Some studies (Singh et al., 2000; Toporcov et al., 2015) have reported that exposure to carcinogens, such as alcohol or tobacco, is more frequent and of longer duration in men than in women, so this ratio may represent a natural sex distribution of LSCC. Another difference observed in our report was the lower proportion of supraglottic cancer among younger patients. A case-control study on LSCC conducted by De Stefani et al. (2004) showed that both tobacco and alcohol were associated with significantly higher risks for supraglottic cancer than for glottic cancer. Patients with supraglottic carcinoma who had smoked for more than 50 years demonstrated a high odds ratio of 46.4 compared to those with glottic cancer. Stefani et al.’s results strongly suggest that supraglottic cancer and glottic cancer are distinct epidemiologic entities, especially when the duration of carcinogen exposure is taken into account. The lower proportion of supraglottic cancer among younger patients in our study may therefore indirectly reflect different risk factors between age groups.

Several studies have reported that young patients with LSCC presented with more advanced disease than older patients, and that survival rates were equal or even lower for younger patients compared with older patients (Nachalon et al., 2018; Singh et al., 2000; Shvero et al., 1987). A retrospective review (Nachalon et al., 2018) covering 160 patients (13 of whom were under 40 years old) revealed that eight younger patients (62%) had stage III and IV cancers vs. 49 (33%) in the older group; the 5-year OS was 69% for younger patients and 90% for older patients. When stratified for early or late stage cancer, there was no significant difference in survival rate between the age groups. The clinical records of 570 patients with LSCC were reviewed by Shvero et al. (1987), including 20 patients (2.8%) aged 40 or younger. Their findings have shown that a higher percentage of younger patients presented with advanced disease, but their survival rates were the same as or compared favorably with survival rates in older patients. A retrospective study (Singh et al., 2000) was carried out over a 9-year period with 209 patients with LSCC, of whom 20 (10%) were under 40 years old. Stage at presentation was similar for all age groups, but the survival rate was significantly lower for younger patients compared with the standard age group. These previous studies have not been able to explain the advanced disease stage and poor survival of younger patients. It has been suggested that younger patients may ignore early symptoms and therefore have later detection (Nachalon et al., 2018), and that the increased aggressiveness of cancer in younger patients may reflect a higher susceptibility to mutagen-induced chromosomal damage (Singh et al., 2000). However, only a very few of these studies were matched studies or contained high enough patient numbers to permit meaningful statistical analysis; statistical bias was inevitable because of the extremely small numbers of younger patients. Misono et al. (2014) reviewed the SEER database and reported 10,429 patients with localized laryngeal cancer who were treated from 1995 to 2009. Ages were classified into four levels (20–39, 40–59, 60–79, and ≥80). The patients aged 20–39 had better survival rates, and the risk of death became higher with increasing age. A review of 99 LSCC patients younger than 30 showed that survival was good, at nearly 90% (Rutt, Hawkshaw & Sataloff, 2010), which is consistent with our findings. In our study population, the stage at presentation (except for M status) was similar for each age group, which means that older patients had higher rates of distant metastasis and tended to have more aggressive disease. As to survival outcome, we examined CSS in an attempt to reduce the impact of other medical comorbidities. Patients in the younger group had a distinctly better survival rate than patients in the older group (5-year CSS: 88.8% vs. 67.6%). When matched for tumor stage, younger patients still achieved better outcomes than older patients at every stage. It is therefore clear that age is an independent prognostic factor in LSCC, and the AJCC staging parameters appear to have less prognostic significance in young patients, with even late-stage cancers indicating a good outcome. It is really interesting to see that young patients who received surgery plus postoperative radiation in stage III disease had relatively poor survival (5-year CSS: 66.7%), even though the difference was not significant when comparing to other modalities (Fig. 4). Due to the limitation of sample size and SEER data base, we were unable to explore the reason. However, the result further indicated that staging parameters appear to have less prognostic significance in young patients.

Given the substantial discrepancies in survival outcome between the age groups, the appropriate management of patients under the age of 40 should be the focus of great attention, especially for locally advanced cancers. The American Society of Clinical Oncology 2006 clinical practice guidelines stated that the LP approach results in equal survival compared with primary surgery for most patients with T3 or T4 disease (Pfister et al., 2006). Study 91-11 by the radiation therapy oncology group (Forastiere et al., 2003) demonstrated that concurrent cisplatin/radiation achieved high rates of LP. Thus, treatment with LP has gained widespread use and is a viable alternative to TL.

However, despite the decrease in survival for patients with laryngeal cancer over the past 20 years (Hoffman et al., 2006), many studies have reported improved survival for patients who undergo TL for advanced stage LSCC. Grover et al. (2015) used the National Cancer Database to identify 969 patients with T4a LSCC who received definitive treatment with either TL plus adjuvant therapy or LP-chemoradiation (LP-CRT). LP-CRT had inferior OS compared with TL (HR = 1.31, 95% CI [1.10–1.57]) and with inverse probability of treatment-weighted model (HR = 1.25, 95% CI [1.05–1.49]). An observation cohort study (Dyckhoff, Plinkert & Ramroth, 2017) that included 107 patients with T4 tumors showed significantly worse OS after LP-CRT compared with TL (HR = 2, 95% CI [1.04–3.7]). Similarly, our study population showed significantly worse 5-year CSS after RT (42.3%) compared with TL (56%) among older patients with T4 tumors. However, this did not occur in the younger cohort, for whom treatment with surgery and/or RT produced excellent survival outcomes at every disease stage. Even with T4 tumors, the 5-year CSS was 90% for RT and 91.5% for TL, with no significant difference (*p* = 0.99). Taking into account the vital function of the larynx, our study strongly suggests that younger patients coped well with all the definitive therapies, and that less aggressive treatment to primary lesions, such as the LP method, can therefore be chosen in preference to TL.

In the current study, only the N, M, and TNM parameters significantly affected survival for younger patients in multivariate analyses. As only two patients had distant metastasis, degree of lymph node involvement seems to be the most important independent prognostic factor. A study (De Paula et al., 2009) that evaluated 724 patients with primary HNSCC also found that cervical metastasis was the only predictor of survival among young patients, suggesting that treatment of HNSCC with an appropriate neck dissection could reduce mortality rates. In our study population, patients in the younger group with advanced N stage (N2 + N3) had higher proportions of poorly differentiated or undifferentiated tumors and supraglottic involvement. This phenomenon coincided exactly with the special biological features of supraglottic cancer. Owing to the wide distribution of lymphatic plexus in the supraglottis (Patel et al., 2018), supraglottic LSCC is the most common cause of cervical metastasis associated with LSCC, and patients with supraglottic LSCC often present with advanced disease characterized by bilateral cervical lymphadenectasis (Patel et al., 2018).

Our study had several limitations. It was a retrospective analysis based purely on SEER database. SEER is unable to report on personal habits and lifestyle that may influence cancer incidence. Furthermore, the adverse feature like positive margins, perineural invasion or vascular invasion after primary surgery was not existent in SEER database. Some variable contained “unknown” category, which could introduce statistical bias. However, our findings heighten the awareness of the clinicians and encourage further studies related to LSCC in young patients.

## Conclusion

LSCC in younger patients is likely to present less aggressive tumor biology than in older patients. Patients in the younger group had significantly better survival rates than those in the older group. Advanced nodal involvement was the most important independent prognostic factor for younger patients. The LP method can be chosen over TL as the preferred strategy for younger patients in cases of locally advanced disease.

## Supplemental Information

10.7717/peerj.7368/supp-1 Supplemental Information 1 Raw data.

10.7717/peerj.7368/supp-2 Supplemental Information 2 Data selection process.

10.7717/peerj.7368/supp-3 Supplemental Information 3 Multivariate Cox regression analysis predicting cancer-specific survival (CSS) for the whole cohort.

## Additional Information and Declarations

### Data Availability

The following information was supplied regarding data availability: SEER data for 1973 to 2015 were obtained from the SEER 18 Registry, including Hurricane Katrina-impacted cases (www.seer.cancer.gov; SEER*Stat version 8.3.5, Database: Incidence-SEER 18 Regs Custom Data (with additional treatment fields), Nov 2016 Sub (1973–2015 varying).
