# Supplementary figures and images for: Studying the impact of young age on prognosis and treatment in laryngeal squamous cell carcinomas using the SEER database
Source: PeerJ. 2019 Jul 25;7:e7368. doi: 10.7717/peerj.7368 (PMC6661153; doi:10.7717/peerj.7368)

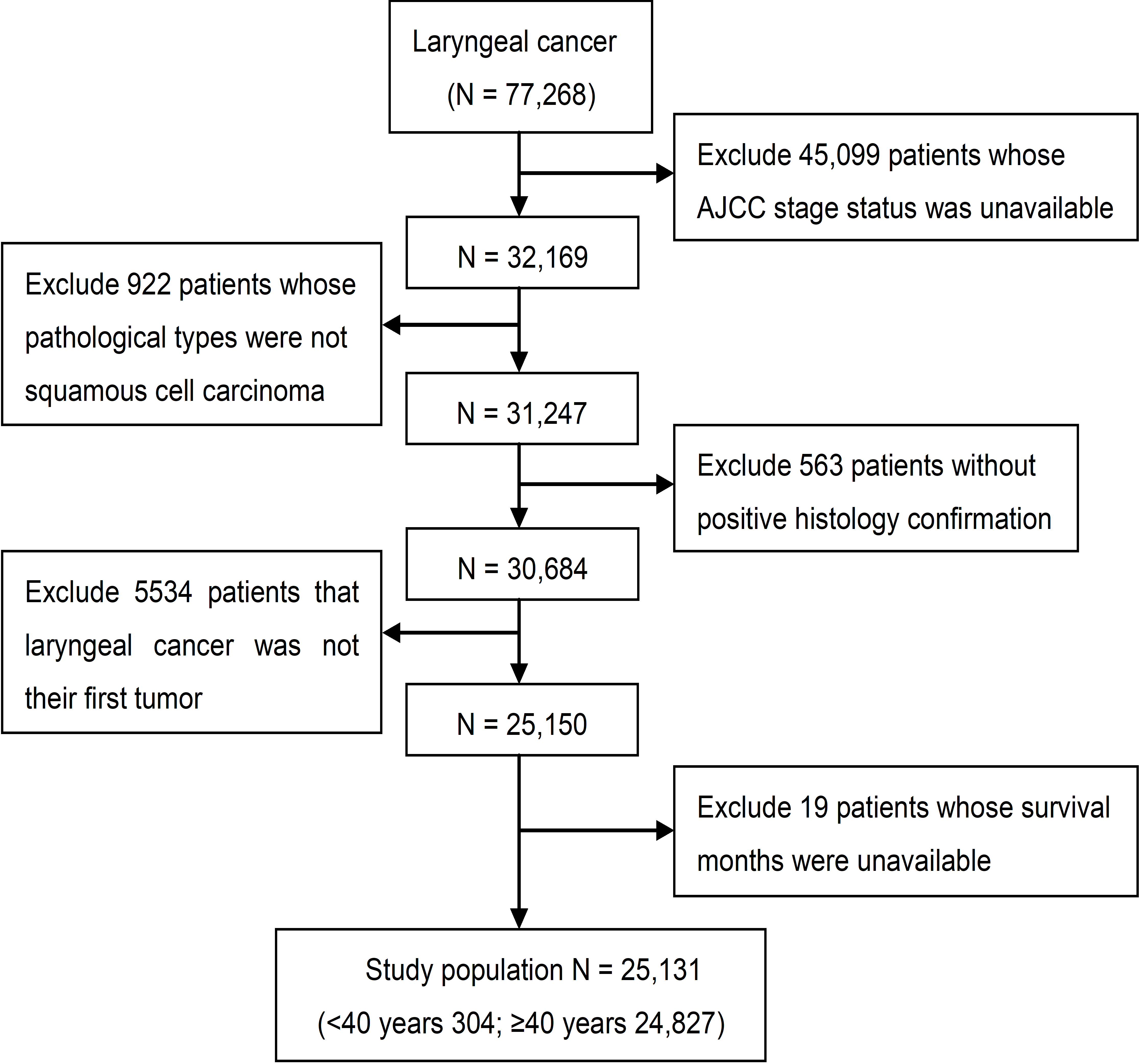

Supplement: Supplemental Information 2 [file peerj-07-7368-s002.png]
