# Supplementary material for: Studying the impact of young age on prognosis and treatment in laryngeal squamous cell carcinomas using the SEER database
Source: PeerJ. 2019 Jul 25;7:e7368. doi: 10.7717/peerj.7368 (PMC6661153; doi:10.7717/peerj.7368)
Supplement: Supplemental Information 3 [file peerj-07-7368-s003.docx]

**Supplementary Table 1.** Multivariate Cox regression analysis predicting cancer-specific survival (CSS) for the whole cohort

| Variable | Whole cohort | |
| --- | --- | --- |
|  | HR (95% CI) | *p* |
| Sex |  | <0.0001 |
| Female/Male | 0.843 (0.789-0.901) |  |
| Age |  |  |
| ≥40/<40 | 3.579 (2.434-5.263) | <0.0001 |
| Race |  |  |
| Black/White |  | 0.645 |
| Others/Black |  | 0.330 |
| Grade |  |  |
| Moderately/Well | 1.263 (1.150-1.388) | <0.0001 |
| Poorly or undifferentiated/Well | 1.525 (1.376-1.690) | <0.0001 |
| Unknown/Well | 1.173 (1.053-1.305) | 0.004 |
| Site |  |  |
| Glottis/Supraglottis | 0.702 (0.656-0.752) | <0.0001 |
| Others/Supraglottis | 1.177 (1.088-1.272) | <0.0001 |
| Stage |  |  |
| Late (III+IV)/Early (I+II) | 1.941 (1.754-2.147) | <0.0001 |
| T status |  |  |
| Late (T3+T4)/Early (T1+T2) | 1.244 (1.151-1.345) | <0.0001 |
| N status |  |  |
| Late (N2+N3)/Early (N0+N1) | 1.555 (1.454-1.662) | <0.0001 |
| M status |  |  |
| M1/M0 | 2.646 (2.359-2.968) | <0.0001 |
| Surgery |  |  |
| No/Yes | 1.285 (1.210-1.364) | <0.0001 |
| Radiotherapy |  |  |
| No/Yes | 1.12 (1.023-1.225) | 0.014 |
| Chemotherapy |  |  |
| Yes/No or unknown | 0.887 (0.829-0.950) | 0.001 |
| Insurance status at diagnosis |  |  |
| Any/None or unknown |  | 0.131 |
| Marital status at diagnosis |  |  |
| Any/None or unknown | 0.746 (0.708-0.786) | <0.0001 |

HR, hazard ratio; CI, confidence interval
